# Case Report: Asymmetric and hierarchical control in world-record rope skipping—biomechanical insights into extreme speed performance

**DOI:** 10.3389/fpsyg.2025.1697856

**Published:** 2025-11-19

**Authors:** Jian-guo Kang, Yufeng Liu, Min Hao, Gongbing Shan

**Affiliations:** 1 Department of Physical Education, Xinzhou Normal University, Xinzhou, China; 2 Department of Mathematics, Xinzhou Normal University, Xinzhou, China; 3 Biomechanics Lab, Department of Kinesiology, Faculty of Arts & Science, University of Lethbridge, Lethbridge, AB, Canada

**Keywords:** 3D motion capture, ground reaction force, biomechanical modeling, accuracy of rope clearance, fatigue, three temporal phases, motor control adaptation

## Abstract

This case-study investigated the motor control strategies underpinning a world-record performance in short-duration, high-intensity rope skipping, focusing on control accuracy and temporal adaptations under extreme speed and fatigue. Methods included 3D motion capture, ground reaction force measurements, and biomechanical modeling. Biomechanical parameters were quantified to identify time-dependent changes and distinctive control strategies. Results demonstrated exceptional clearance accuracy, with left foot operating at mechanical limit and right providing a safety margin. Progressive adaptations of COG height and ROM reflected fatigue-related adjustments, revealing a three-phase adaptation model. Kinetic analyses indicated a functional division of labor, with left leg serving as the power limb and right as the timing limb. Kinematic findings further identified a hierarchical control strategy: trunk adjustments provided rhythmic scaffolding, left leg joints stabilized during contact, and right leg joints enhanced clearance mobility. These results illustrate how asymmetric, hierarchical coordination optimizes performance under extreme temporal and spatial constraints.

## 1 Introduction

The single rope speed sprint—often called the run-in-place jump rope technique—is a dynamic variant of traditional rope skipping. Unlike the conventional two-foot hopping style, it alternates foot contacts in a rapid running motion while remaining in place. Performed on the balls of the feet, it demands agility, coordination, and precise timing to synchronize limb movements with continuous rope rotations. Its biomechanical and physiological demands make it a staple in both general fitness and elite athletic training (Trecroci et al., 2015; Orhan, 2013; Zhou et al., 2025; Pratama et al., 2018), enhancing speed, neuromuscular coordination, and conditioning.

Within competitive jump rope, the 30-s single rope speed sprint is a core speed event sanctioned by the International Jump Rope Union (IJRU, 2024). It is a demanding test of human movement, combining intense physiological and neuromechanical challenges.

Physiologically, the event belongs to short-duration, high-intensity activities (10–90 s), dominated by anaerobic metabolism (Wilmore et al., 2004; Spurway, 1992; Wells et al., 2009; Sahlin, 2014). Energy supply shifts from the alactic anaerobic system (up to ~10 s) to the lactic anaerobic system (10–60/90 s). After the initial 10 s, depletion of intramuscular adenosine triphosphate (ATP) activates the lactic pathway, leading to lactic acid accumulation and muscle fatigue. This predominates when oxygen delivery cannot meet metabolic demands—such as in the 30-s rope speed sprint—allowing sustained high output without aerobic reliance.

Neuromechanically, athletes must maintain precise limb coordination while combating rapid fatigue, pushing the limits of motor control and short-term endurance (Zhou et al., 2025; Lorke et al., 2022; Bruce et al., 2023; Goto et al., 2021). Performance is measured by counting rope passes beneath one foot over 30 s and doubling the result.

Xiaolin Cen, an elite Chinese athlete, holds the current world record with 232 jumps in 30 s (IJRU, 2025). To the best of our knowledge, there is a paucity of biomechanical research on this specific technique. Given its extreme cyclic frequency, technical precision, and fatigue dynamics, the event offers a rare opportunity to investigate motor control at the performance stabilization stage in a mature elite athlete. Biomechanics research in human movement aims to enhance performance and reduce injury risk (Hay, 1993; Shan et al., 2015; Zatsiorsky, 2008; Ballreich and Baumann, 1996). Quantifying performance through biomechanical analysis yields objective indicators, strengthening evidence-based coaching (Lees, 1999; Shan et al., 2019; Carling et al., 2008; Curran and Frossard, 2012). For elite athletes operating near technical limits after years of practice, such analysis can reveal subtle technical adaptations over time (Cavanagh and Hinrichs, 1981; Smith, 2003; Issurin, 2013). Cen’s performance enables a data-driven examination of plateau-level performance under authentic competitive conditions, with insights relevant to other sports requiring rapid, repetitive, and precisely coordinated movements, such as sprint cycling, speed skating, and high-cadence running. These findings can inform future research and training design, advancing coaching science.

Therefore, this case study aims to address the following research questions:

1. What are the distinctive biomechanical and motor control strategies employed by the reigning world record holder during the 30-s single-rope speed sprint?
2. How do these strategies temporally adapt to progressive fatigue under conditions of extreme speed and physical demand?

Grounded in Cen’s ongoing performance progression, the study seeks to provide biomechanical evidence to inform models of motor control and skill optimization, offering direct implications for athlete development and high-performance coaching.

## 2 Case description

This case study examined Xiaolin Cen, a 23-year-old elite jump rope athlete from Guizhou Province, China, and a current student at Guangzhou Polytechnic of Sport. Cen holds multiple world records, including the Single Rope Speed Sprint (in 30 s) and Single Rope Speed Endurance (in 180 s) (IJRU, 2025), and has previously held Guinness World Records double under event (Guinness, 2019). His most recent performance—232 jumps in 30 s at the 2025 IJRU World Championships—surpassed his own 2023 record of 228 jumps (XinhuaNet, 2023).

Cen’s competitive record illustrates not only extraordinary physical capacity but also exceptional motor control under extreme temporal and physiological constraints. To better understand the mechanisms supporting such elite performance, the biomechanical data analyzed in this case report were collected in 2024 during a controlled laboratory simulation conducted between Cen’s official world-record performances in 2023 (228 jumps) and 2025 (232 jumps). Although the data were not recorded during the competition itself, the test captured the athlete’s peak performance capacity within this period, thereby providing a representative and valid basis for elucidating the motor control strategies underpinning his world-record achievements.

Cen’s competitive career (BaiduBaike, 2025) began in primary school, where he entered the sport at the age of nine and rapidly advanced through specialized training after joining his school’s jump rope team in 2012. By 2015, at the age of 14, he had already captured gold in the 30-s single rope speed event at the Asian Jump Rope Championships and set multiple world records at the inaugural World Inter-School Rope Skipping Championships. Over the past decade, he has repeatedly broken both his own records and those of other world-class competitors, demonstrating unparalleled consistency and progression in high-speed rope skipping performance.

Cen’s competitive record illustrates not only extraordinary physical capacity but also exceptional motor control under extreme temporal and physiological constraints. His repeated ability to improve performance at the highest level indicates a uniquely effective combination of biomechanical efficiency, technical refinement, and adaptive motor strategies. Given his status as the fastest recorded rope skipper in history, Cen represents a rare and highly valuable subject for scientific investigation. Studying the temporal evolution of his motor control during record-setting performance can yield insights into skill optimization, fatigue management, and coordination adaptation, thereby informing both theoretical frameworks in motor control and applied practices in movement science, coaching, and athlete development.

## 3 Methods

### 3.1 Subject

The participant was Xiaolin Cen, a 23-year-old elite jump rope athlete (body mass: 53.9 kg; height: 1.65 m). At the time of testing, he had no injuries in the previous 6 months, had not engaged in strenuous exercise within the preceding 24 h, and was in good health and athletic condition. The test protocol was approved by the host university’s ethics committee. Prior to participation, the subject was fully informed about the study procedures, provided written consent, and voluntarily took part in the experiment. Testing was conducted using his own competition rope. Before the test, he completed a self-directed warm-up lasting approximately 40 min, which included jogging, stretching, and moderate-speed jump rope exercises to attain a state of competition readiness.

### 3.2 Synchronized data collection and biomechanical modeling

Movement data were collected using two synchronized systems:

1. A 13-camera 3D motion capture system (VICON V5, Oxford Metrics Ltd., Oxford, UK) with reflective markers (diameter = 9 mm) sampled at 300 Hz.
2. Four force plates (Kistler, Model 9286BA, Winterthur, Switzerland) sampled at 1,500 Hz (i.e., five times the motion capture frequency).

The jump rope was performed on a platform integrating the four force plates, ensuring ground reaction force (GRF) collection for every step. In total, 45 reflective markers were applied (Zhou et al., 2025): 39 on the subject’s body and 6 (3M flex-tape) on the rope. The 39 body markers were positioned at specific anatomical landmarks, including the left and right temporal regions, left and right posterior head regions, sternal end of the clavicle, xiphoid process of the sternum, C7 and T10 vertebrae, right scapula, left and right anterior superior iliac spines, posterior superior iliac spines, right and left acromia, lateral sides of the upper arms, lateral epicondyles, lateral forearms, styloid processes of the radii and ulnae, distal ends of the third metacarpal bones, lateral sides of the thighs and shanks, lateral tibial condyles, lateral malleoli, calcanei, and halluces (big toes). Extensive calibration procedures ensured an accuracy of <1 mm, following the manufacturer’s guidelines.

Based on marker placement, a validated anthropometry-based 15-segment biomechanical model was constructed (Zhou et al., 2025; Shan and Bohn, 2003; Zatsiorsky, 2002; Winter, 2009), comprising the head, trunk (upper and lower), upper arms, lower arms, hands, thighs, shanks, and feet. This model has been previously applied to various sports skills (Shan and Westerhoff, 2005; Shan, 2008; Penitente et al., 2011; Zhang et al., 2016; Liu et al., 2020).

As a case study focusing on motor-control pattern identification, this research deliberately centered on kinematic parameters to illustrate the key control mechanisms underlying extreme-speed performance. These parameters included joint angles, joint ROM, and whole-body Center of Gravity (COG) trajectories derived from biomechanical modeling. In addition, variations in these kinematic parameters were examined in relation to jump impulse (i.e., kinetics) obtained from GRF measurements. This analytical framework provided valuable insights into the mechanisms governing motor control during speed performance (Shan et al., 2011).

The combination of 13 cameras, small markers, and four force plates provided the subject considerable freedom of movement within the capture volume, allowing him to perform with his trained “motor control style.”

**Figure 1:**
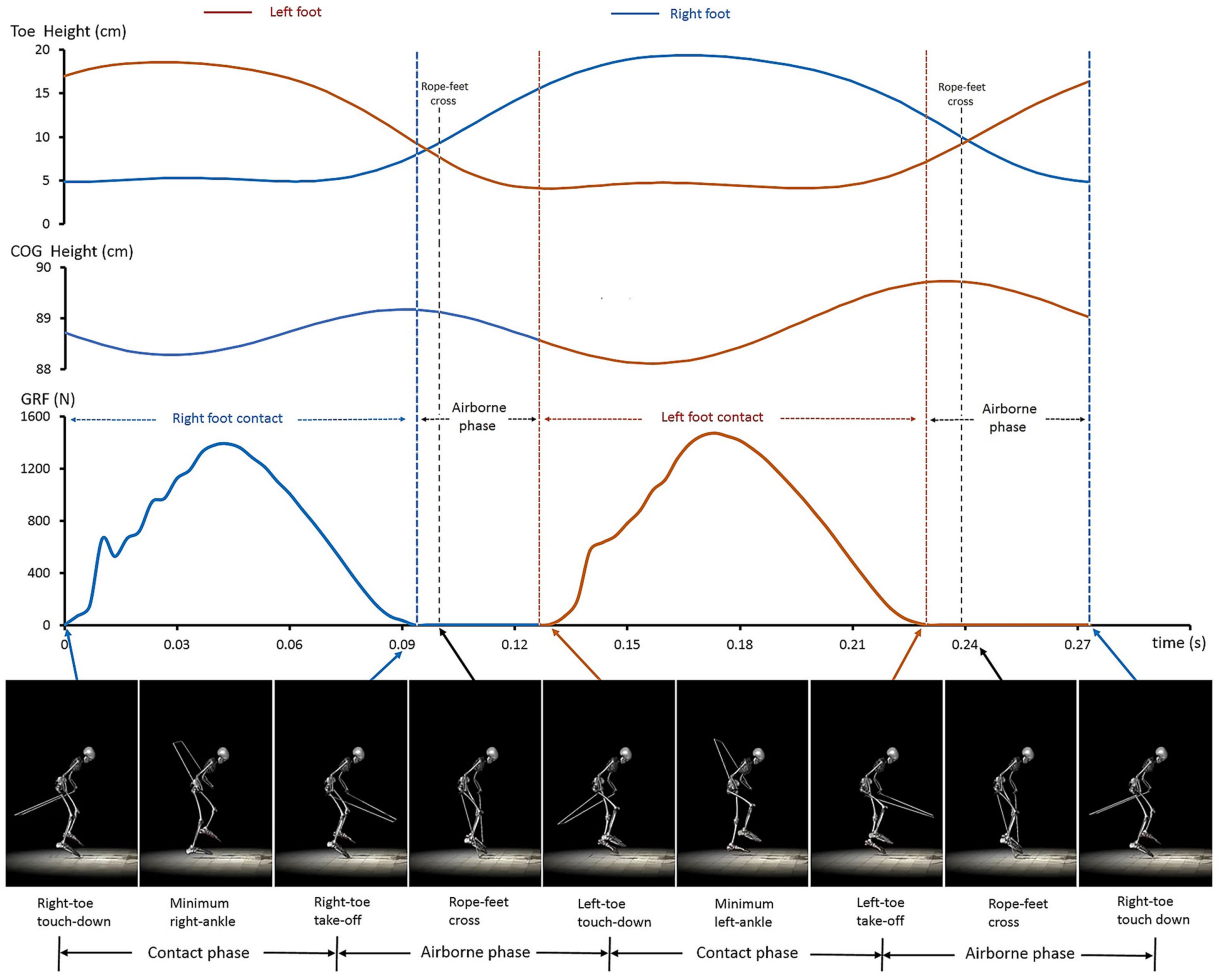
A representative one-cycle dataset obtained through synchronized measurements and biomechanical modeling. The figure illustrates the main characteristics of body–rope–ground interactions (graphs) and joint coordination quantified by biomechanical modeling (bottom).

Figure 1 presents a representative cycle of synchronized measurements, showing dynamic changes in toe height, the athlete’s COG, and GRF, along with the 15-segment biomechanical model, as well as the phases and key time events within the cycle.

### 3.3 Data processing, parameter selection, and data analysis

The synchronized measurements and biomechanical modeling produced a wide range of kinematic and kinetic parameters. From the full dataset, only those relevant to the study’s objectives were selected to ensure focused reporting (Zhang et al., 2016; Liu et al., 2020; Kowalski et al., 2018; Thomas et al., 2022; Shan, 2022). These included:

- Kinematic parameters related to control and COG changes,
- Toe and rope–feet crossing height,
- Cycle time change over 30 s,
- Foot contact time change over 30 s,
- GRF and jump impulse change over 30 s.

To evaluate temporal adaptations in coordination under extreme speed and fatigue, trendline analyses (Moore et al., 2013) were conducted. Because human motor control is inherently nonlinear (Magill, 2001; Stergiou, 2018; Stergiou and Decker, 2011), second-order polynomial trendlines were applied using the trendline analysis function in SPSS. Following established practice (Moore et al., 2013), parameters with *R*^2^ > 0.5 were considered to demonstrate meaningful time-dependent changes. Further, parameters, showing differences between left- and right-side controls, were also selected for analyses. Accordingly, only parameters meeting these criteria were analyzed in the case report. Where clear time-based changes were evident, the 30-s trial was divided into sections, and one-way ANOVA followed by a Scheffe *post-hoc* test was applied to compare differences across sections.

All statistical analyses were conducted using IBM SPSS Statistics 23 (IBM Japan, Tokyo, Japan). Statistical significance was set at *p* < 0.05.

## 4 Results and discussion

**Figure 2:**
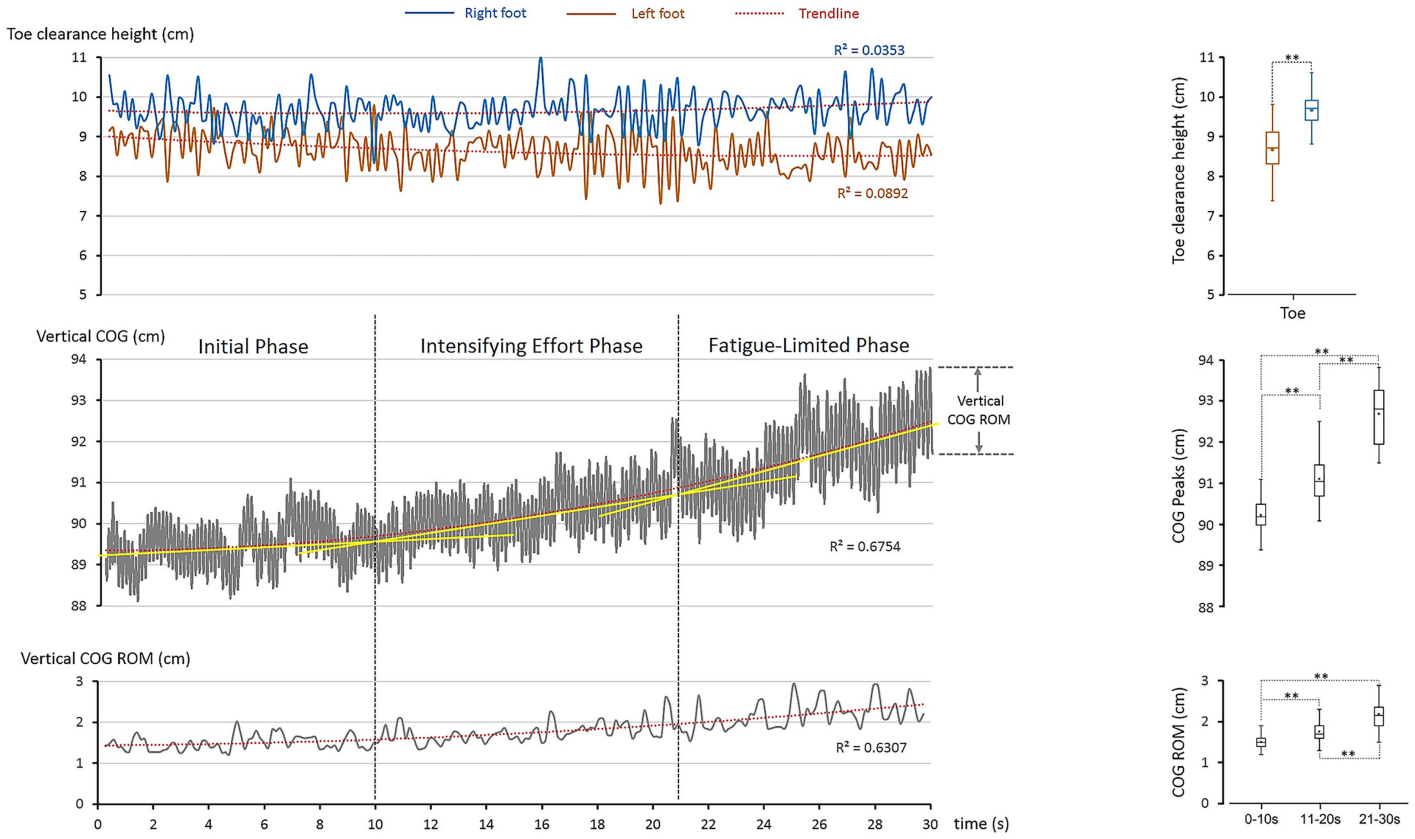
Foot clearance and whole-body control (COG) during the 30-s single-rope speed sprint. The *left column* illustrates the temporal excursions of selected parameters, while the *right column* presents their statistical distributions. Boxplots display the interquartile range (box), median (horizontal line), mean (dot), and range (whiskers: minimum–maximum). Dashed lines with asterisks denote statistically significant differences between conditions (*p* < 0.05, significant; *p* < 0.001, highly significant). *Top panel*: toe clearance at rope–foot crossing showed stable but asymmetric patterns between limbs (trendline analysis: *R*^2^ < 0.089), confirmed by significant differences (**: *p* < 0.001). *Middle panel*: vertical COG increased nonlinearly over time (trendline analysis: *R*^2^ = 0.675). The upward drift was segmented into three phases using tangent-line approximations (yellow lines in the Figure). These tangent-based divisions were visually determined from the second-order polynomial curve, reflecting notable changes in the rate of COG elevation over time. Phase comparisons revealed significant differences (**: *p* < 0.001). *Bottom panel*: Vertical COG ROM also rose progressively (trendline analysis: *R*^2^ = 0.631) with significant phase differences (**: *p* < 0.001).

The laboratory-based data collection yielded a total of 207 jump cycles within the 30-s performance interval, corresponding to an average jump frequency of approximately 7 Hz. This quantitative profile provides a foundation for assessing the distinctiveness of the elite performance. Among the selected parameters, two features emerged as particularly informative in characterizing the motor control strategies of the world-record holder: (1) accuracy and asymmetry of foot control (Figure 2, top panel), and (2) progressive whole-body adaptations represented by vertical COG regulation (Figure 2, middle and bottom panels).

### 4.1 Exceptional accuracy and asymmetric motor control

#### 4.1.1 Control accuracy

The toe clearance height (toe height at rope-foot cross) highlighted the athlete’s remarkable precision under extreme temporal constraints. For the left foot, average clearance above the baseline toe marker (5 cm, Figure 1) was 3.7 cm (8.7–5.0 cm). At its minimum, clearance narrowed to 2.3 cm (7.3–5.0 cm), leaving an extremely small safety margin. Such precision is inherently risky, as even minimal deviation could result in rope contact. Yet, the athlete consistently sustained this clearance across the 30-s sprint, reflecting finely tuned neuromuscular coordination developed through long-term elite training (Smith, 2003; Schmidt et al., 2018; Sim and Kim, 2010).

For the right foot, average clearance was 4.7 cm (9.7–5.0 cm). Even at its minimum (8.3 cm), clearance remained 3.3 cm, providing a slightly safer buffer than the left. Variability was low on both sides (SD = 0.5 cm left; 0.4 cm right), demonstrating stable accuracy despite the extreme repetition rate.

#### 4.1.2 Control asymmetry

Asymmetry is a foundational aspect of human movement, evident in everyday activities such as gait and accentuated in high-performance sports (Afonso et al., 2020; Przybyla et al., 2012; Panzer et al., 2021). In this case, clear inter-limb asymmetry was observed (*p* < 0.001). The right foot consistently maintained greater rope-foot clearance, while the left foot operated close to the mechanical limit. We interpret this asymmetry as a reflection of functional specialization: the left foot optimized efficiency at the edge of risk, while the right foot compensated with additional clearance to reduce error likelihood. Such limb-specific adaptation reflects a finely balanced strategy between risk and safety to sustain performance at record-level speed. The observed coordination pattern aligns with Bernstein’s degrees of freedom problem (Latash, 2014; Turvey et al., 2014) and Latash’s synergy framework (Latash, 2012), which describe how skilled movement emerges through motor learning and training. As Bernstein proposed, the nervous system gradually organizes redundant degrees of freedom, “freezing” and later “freeing” them to achieve efficient coordination. Consistent with Latash’s view, the asymmetrical limb roles exemplify the functional use of motor redundancy, forming an inter-limb synergy that sustains precision, adaptability, and efficiency under extreme temporal and spatial constraints.

### 4.2 Progressive adaptations of whole-body coordination

Figure 2 also illustrates the vertical COG trajectory (middle panel) and cycle-to-cycle COG ROM (bottom panel). The nonlinear upward drift of the COG trendline revealed systematic adjustments with time (*R*^2^ = 0.675) as fatigue accumulated. Approximating the trendline with tangent lines provided a robust basis for segmenting performance into three temporal phases, each aligned with distinct physiological energy systems and motor-control adaptations.

#### 4.2.1 Phase I—Initial Phase (0–10 s)

The tangent line was nearly flat, showing negligible upward drift. Average peak COG height was 90.2 ± 0.4 cm, and average COG ROM was 1.5 ± 0.2 cm. These values reflect precise control of vertical displacement, sustained primarily by the alactic anaerobic system (Wilmore et al., 2004; Spurway, 1992; Wells et al., 2009; Sahlin, 2014). The athlete preserved rhythm and clearance with minimal energetic cost.

#### 4.2.2 Phase II—Intensifying Effort Phase (10–21 s)

The tangent line displayed a moderate slope, indicating accumulating fatigue. Average peak COG height rose significantly to 91.1 ± 0.5 cm (+0.9 cm or 1.0%, *p* < 0.001), while average COG ROM expanded to 1.8 ± 0.3 cm (+16.6%, *p* < 0.001). This adjustment corresponds to reliance on lactic anaerobic metabolism, where accumulating lactate impairs efficiency (Wilmore et al., 2004; Spurway, 1992; Wells et al., 2009; Sahlin, 2014). To sustain clearance, the athlete elevated jump height, accepting greater vertical displacement and reduced energy economy.

#### 4.2.3 Phase III—Fatigue-Limited Phase (21–30 s)

The tangent line steepened sharply, showing a pronounced upward drift. Average peak COG height increased to 92.7 ± 0.5 cm (+1.6 cm or 1.7% from Phase II; +2.5 cm or 2.7% from Phase I, both *p* < 0.001), while average COG ROM rose further to 2.2 ± 0.3 cm (+24.3% vs. Phase II; +44.9% vs. Phase I, both *p* < 0.001). These changes reflect severe fatigue at the physiological limit. Enlarged vertical displacement indicated a biomechanical trade-off—sacrificing efficiency to preserve rope clearance under fatigue.

Taken together, the phase segmentation reflects an integrated temporal adaptation of motor control under extreme performance demands. The three phases—Initial, Intensifying Effort, and Fatigue-Limited—were defined through combined kinematic and kinetic indicators that captured progressive fatigue responses. As illustrated in Figure 2, the nonlinear rise in vertical COG (*R*^2^ = 0.675) served as the primary reference, with tangent-line approximations marking distinct transitions in control dynamics. The Initial Phase (0–10 s) was characterized by stable COG height, ROM, and short contact times, supported by alactic anaerobic energy. The Intensifying Effort Phase (10–21 s) showed moderate increases in these parameters, reflecting the onset of lactic anaerobic activation. The Fatigue-Limited Phase (21–30 s) displayed steep COG elevation, expanded ROM, and longer contact times—signifying compensatory adjustments to sustain clearance as power declined. Collectively, these patterns demonstrate how mechanical and physiological indicators converge to reveal a structured, three-phase model of fatigue adaptation in extreme-speed rope skipping. This phase-specific evolution of control sets the foundation for the subsequent kinetic analyses, which further elucidate how the lower limbs manage force production and timing across fatigue progression.

### 4.3 Kinetic and cycle control

**Figure 3:**
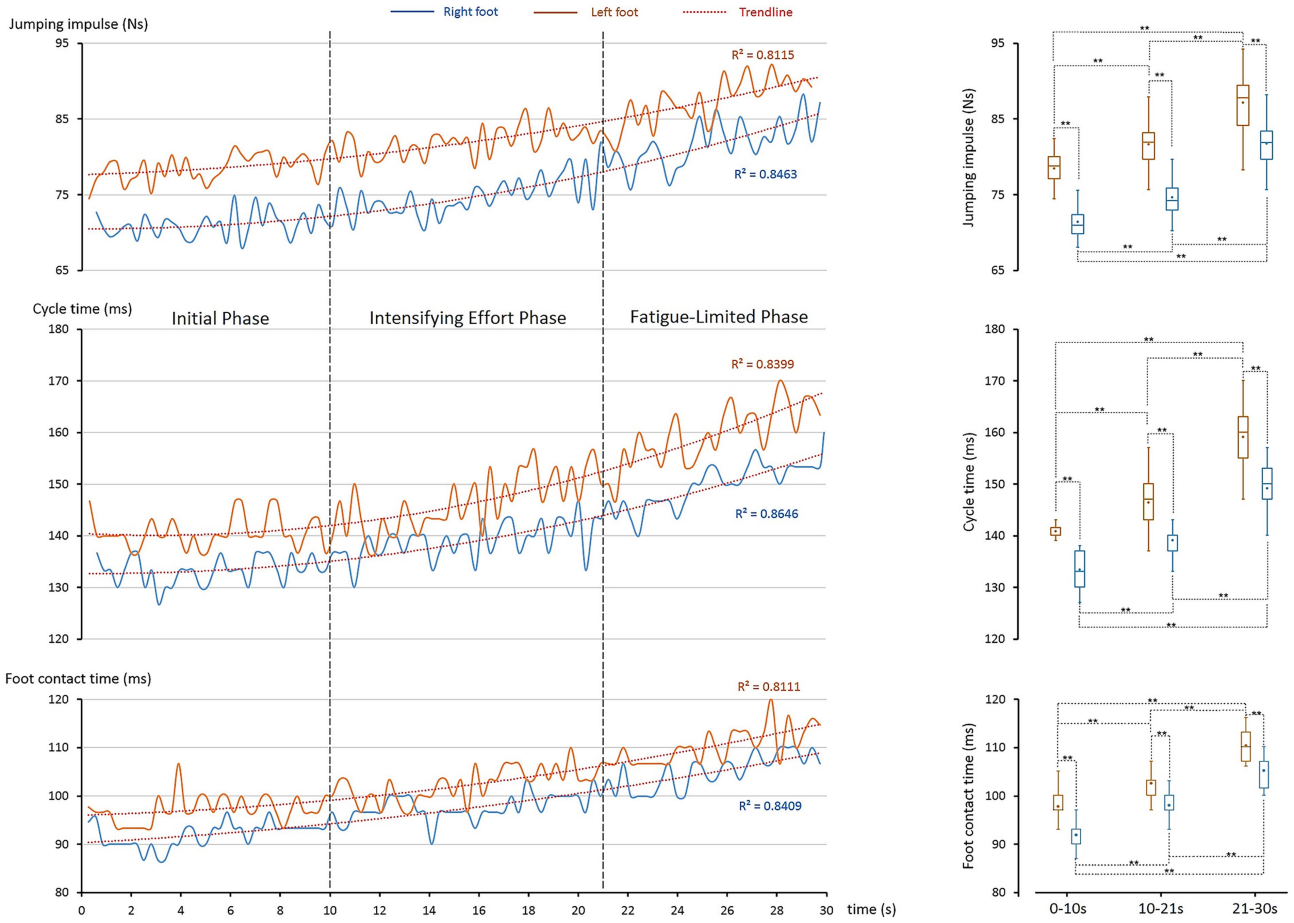
Kinetic adaptations during the 30-s single-rope speed sprint. The *left column* shows temporal changes in key kinetic parameters, while the *right column* presents their statistical distributions. Boxplot elements are as defined in Figure 2. *Top panel*: jumping impulse increased progressively for both feet (trendline analysis: *R*^2^ > 0.812) with significant differences across phases (**: *p* < 0.001). *Middle panel*: cycle time showed similar time-dependent increases (*R*^2^ > 0.840) with phase differences (**: *p* < 0.001). *Bottom panel*: foot contact time also rose progressively (*R*^2^ > 0.811), with significant differences across phases (**: *p* < 0.001).

Building upon the identified temporal phases, the kinetic analysis examines how ground reaction forces and related temporal parameters evolved across the 30-s sprint, revealing phase-dependent adaptations in impulse generation, cycle timing, and foot contact duration that underpin sustained performance under fatigue. Figure 2 showed that the athlete sustained toe clearance through progressive whole-body adaptations and inter-limb specialization. These feats required finely tuned adjustments in force generation and timing. The kinetic and cycle analyses (Figure 3) highlighted three key parameters—jump impulse, cycle time, and foot contact time—that underpinned these control mechanisms.

#### 4.3.1 Adaptations to fatigue and COG control

All three parameters increased significantly with time (*R*^2^ > 0.811).

- Impulse (Figure 3, top panel): left impulse rose 4.1% (Phase I → II), 6.7% (II → III), totaling 11.0%. Right impulse increased 4.5, 9.5, and 14.4%, respectively.
- Cycle time (Figure 3, middle panel): left cycle time increased 4.1 and 8.6%, totaling 13.1%; right side rose 4.3 and 7.3%, totaling 11.9%.
- Foot contact time (Figure 3, bottom panel): left contact extended 5.3 and 7.6% (13.4% total); right increased 6.4 and 7.4% (14.2% total).

These results show progressive adaptations to fatigue, with both limbs systematically altering force and timing to sustain clearance.

#### 4.3.2 Asymmetric kinetic control

Impulse was consistently greater on the left foot (78.5–87.2 Ns vs. 71.5–81.8 Ns, all *p* < 0.001). The reduction in asymmetry from 9.8% (Phase I) to 6.6% (Phase III) suggests a fatigue-induced shift toward a more bilateral control strategy, with an increased relative contribution from the right foot under fatigue. Cycle times also differed consistently (in average: left 141–159 ms vs. right 133–149 ms, p < 0.001), indicating the left side required longer durations to generate vertical force. Similarly, foot contact times remained longer on the left (97–110 ms vs. 92–105 ms, *p* < 0.001).

Together, these findings confirm a division of labor: the left leg served as the power limb, generating vertical displacement, while the right leg functioned as the timing limb, optimizing cadence. This asymmetry provided the kinetic foundation for the clearance strategy described in Section 4.2.

### 4.4 Kinematic adaptations

**Figure 4:**
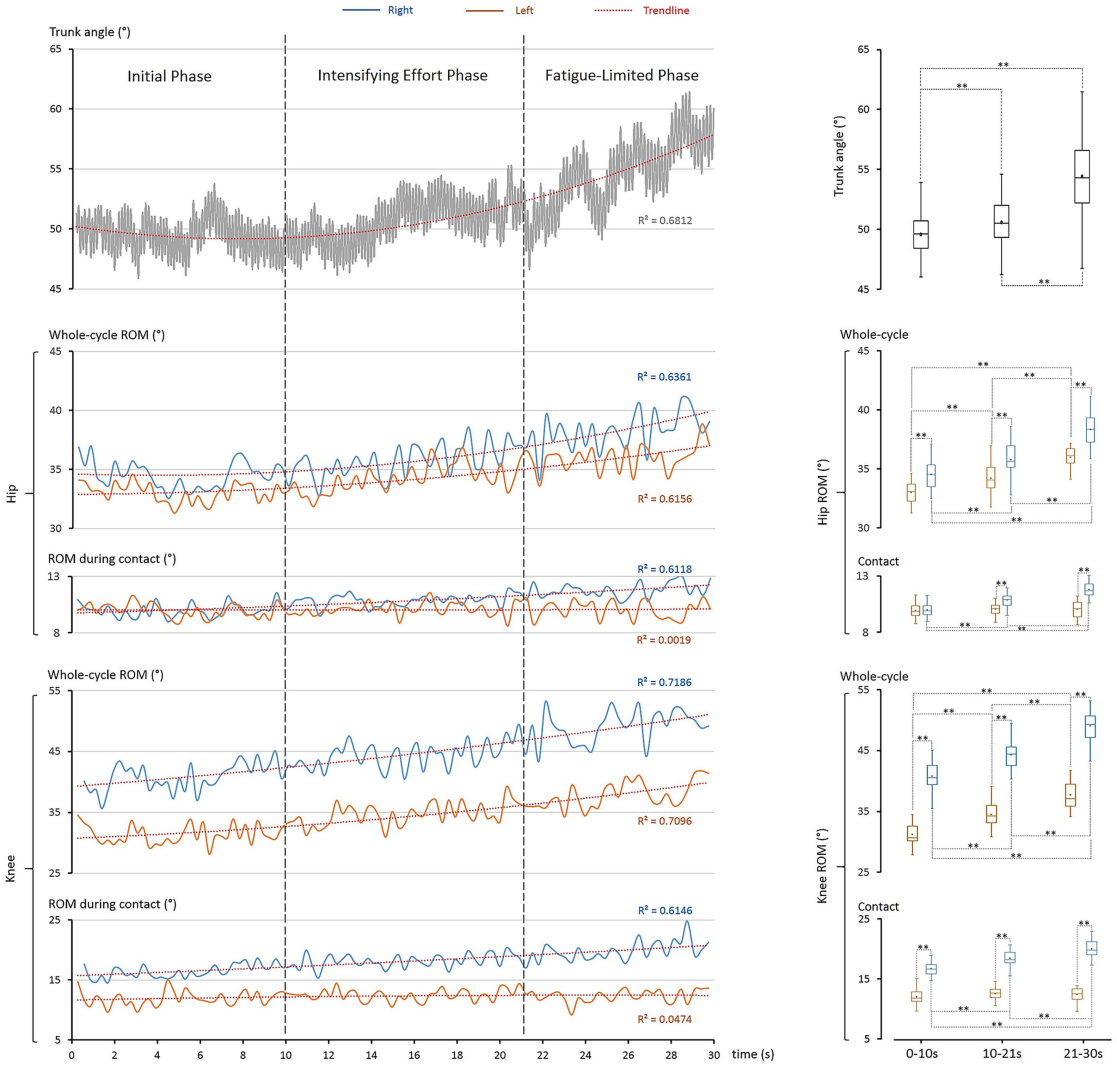
Kinematic adaptations during the 30-s single-rope speed sprint. The *left column* illustrates time-dependent changes in key kinematic parameters, and the *right column* presents corresponding statistical distributions. Boxplot elements are as defined in Figure 2. *Top panel*: trunk angle progressively increased across phases (*R*^2^ = 0.681), with significant differences among phases (**: *p* < 0.001). *Middle panel*: hip ROM increased over time in both limbs (*R*^2^ > 0.616), with the right hip consistently larger (**: *p* < 0.001); left-hip ROM during contact remained stable (*R*^2^ = 0.002), while right-hip increased significantly (*R*^2^ = 0.612, **: *p* < 0.001). *Bottom panel*: knee ROM showed strong asymmetries, with larger values in the right knee (**: *p* < 0.001). Whole-cycle ROM rose progressively (*R*^2^ > 0.709) for both, while right-knee ROM during contact increased significantly (*R*^2^ = 0.615, **: *p* < 0.001) and left-knee ROM remained stable (*R*^2^ = 0.047).

Kinematic adaptations, primarily in the sagittal plane (trunk, hip, knee), further demonstrated hierarchical whole-body coordination (Figure 4). Most parameters showed strong time-dependent changes (*R*^2^ > 0.611). Importantly, asymmetries revealed a structured strategy: the trunk acted as a scaffold, the left leg stabilized during contact, and the right leg enhanced mobility to optimize rope passage.

#### 4.4.1 Trunk: core stability and clearance

Trunk inclination rose progressively (49.5 ± 1.5° → 54.3 ± 3.0°, *p* < 0.001), paralleling COG elevation. The trunk plays a central role in the kinetic chain, enabling the transfer and control of force to distal segments (Kibler et al., 2006; Dendas, 2010; Saeterbakken et al., 2021). Forward lean provided a stable base for rapid footwork and rhythm (Nagahara et al., 2019; Carson et al., 2024; Boyle, 2016), but reduced energetic economy. Consequently, under fatigue, trunk posture became progressively more upright, redistributing joint power—decreasing hip contribution, increasing knee contribution, and leaving ankle contribution unchanged. This phenomenon has also been observed in other sport skills (Vanrenterghem et al., 2008).

#### 4.4.2 Hip: asymmetry and left-side stability

Hip ROM per cycle increased bilaterally (left: +8.9%, right: +10.8%, both *p* < 0.001), with consistently greater right-side increase (4.5–6.4%). During ground contact, the left hip remained stable (10.0–10.1°, *p* > 0.3), while the right hip expanded progressively (10.0 → 11.8°, +18.0%, *p* < 0.001). This division reflects left-side stability under load and right-side mobility enhancement.

#### 4.4.3 Knee: asymmetry and left-side stability

Knee ROM per cycle increased substantially (left: +20.9%, right: +20.5%, both *p* < 0.001), with the right knee consistently larger (16.6–41.6% difference). During ground contact, the left knee remained stable (11.9–12.4°, *p* > 0.05), while the right knee increased markedly (16.4 → 19.9°, +21.2%, *p* < 0.001). Thus, the left knee acted as a stabilizer, while the right knee contributed progressively greater clearance.

#### 4.4.4 Hierarchical integration

**Figure 5:**
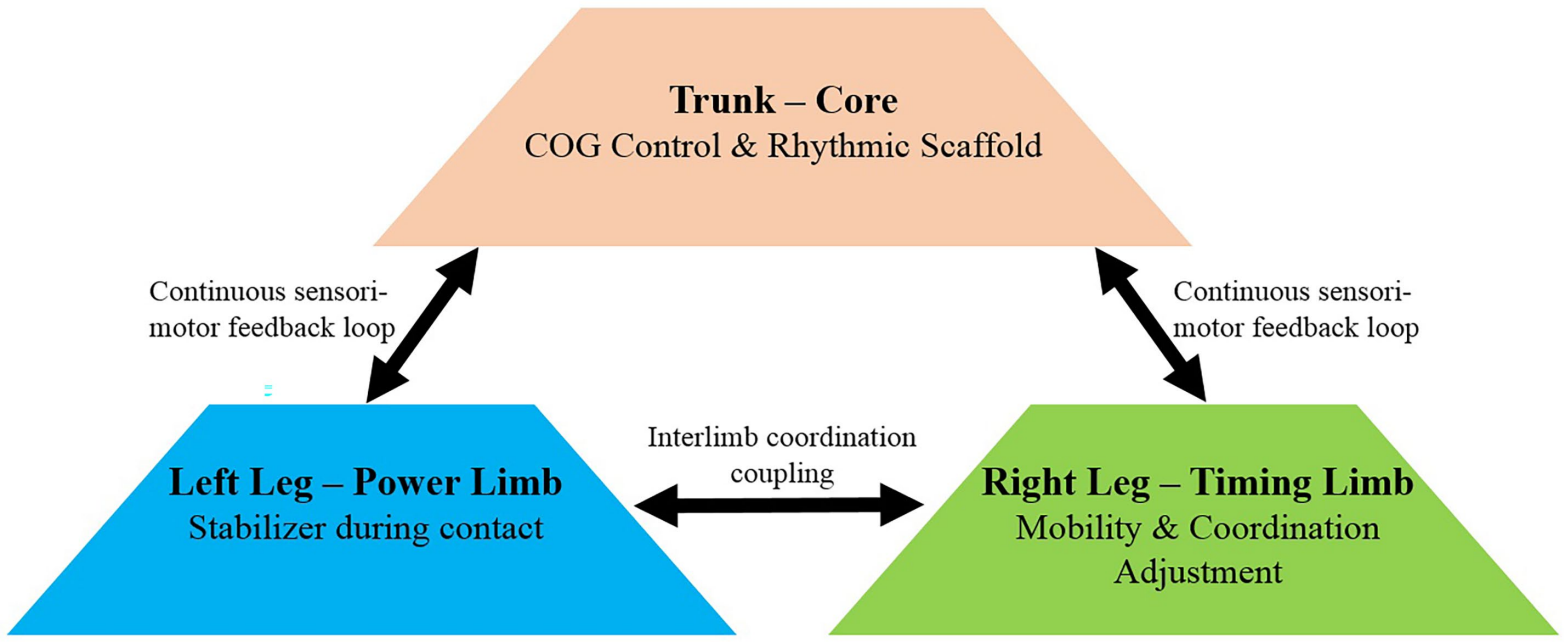
Hierarchical control model illustrating asymmetric coordination during extreme-speed rope skipping.

Taken together, Section 4.4 shows a hierarchical strategy: the trunk provided a rhythmic scaffold, the left hip and knee stabilized contact mechanics, and the right limb enhanced mobility and clearance (Figure 5). This asymmetrical, integrated control optimized rhythm, efficiency, and clearance reliability under record-level performance.

## 5 Practical implications for elite jump-rope training and performance

This case report highlights three core mechanisms underlying record-level rope skipping: precision of clearance, kinetic division of labor, and kinematic hierarchy. Together, these strategies illustrate how an elite athlete adapts to extreme temporal and metabolic constraints.

### 5.1 Accuracy of rope clearance

Segmentation of the COG trajectory into three phases—Initial, Intensifying Effort, and Fatigue-Limited—demonstrates how jump mechanics adapt to escalating metabolic stress. The asymmetric but highly precise toe-clearance strategy highlights the necessity of centimeter-level precision under millisecond timing demands, underscoring the critical risk–reward balance of elite performance. Coaches can apply this framework to monitor athletes’ fatigue responses and train phase-specific adaptations, such as improving efficiency in the early phase and sustaining coordination under fatigue in the later stages.

### 5.2 Kinetic division of labor

The kinetic analysis reveals a consistent functional asymmetry: the left leg operates as the dominant “power limb,” securing sufficient vertical displacement, while the right leg functions as the “timing limb,” fine-tuning cadence and rhythm. This division of labor reflects long-term motor learning and coordination refinement at extreme movement frequencies. Practically, coaches and practitioners could evaluate an athlete’s natural limb asymmetry to identify dominant functional roles. Instead of enforcing perfect bilateral symmetry, training can strengthen each limb’s specialized contribution—enhancing the power limb’s force production and the timing limb’s coordination precision—to optimize efficiency and reduce performance errors under high-speed conditions.

### 5.3 Kinematic hierarchy of control

The kinematic analysis shows a hierarchical control strategy. The trunk provided the rhythmic scaffold, the left hip and knee acted as stabilizers during ground contact, and the right hip and knee enhanced mobility and clearance. This integrated trunk–limb coordination optimized rhythm, efficiency, and performance, enabling sustained clearance despite accumulating fatigue. From an applied perspective, training should emphasize integrated trunk–limb coordination, promoting trunk stability alongside dynamic leg differentiation. Drills combining rhythmic control, core endurance, and alternating unilateral emphasis could reinforce this top-down coordination, thereby improving both efficiency and fatigue resistance in high-intensity cyclic movements.

## 6 Conclusion

This case report highlights how a world-record rope skipper integrated extraordinary precision with an asymmetric, hierarchical control strategy to sustain performance under extreme temporal and metabolic constraints. The athlete maintained millisecond-level timing and centimeter-level clearance through a functional division of labor: the trunk provided a rhythmic scaffold, the left limb acted as a stabilizer during contact, and the right limb enhanced mobility and clearance. Progressive adaptations of force generation, cycle timing, and segmental kinematics reflected the transition from alactic to lactic anaerobic metabolism, revealing how fatigue reshapes coordination. Together, these findings illustrate how long-term motor learning enables elite athletes to balance accuracy, asymmetry, and adaptability in achieving record-level performance.

## Data Availability

The original contributions presented in the study are included in the article/supplementary material, further inquiries can be directed to the corresponding authors.
